# Impact of international experience on research capacity of Chinese health professionals

**DOI:** 10.1186/s12992-014-0086-4

**Published:** 2015-02-07

**Authors:** Tingjiao Liu, Liming Zhang, Lina Sun, Xun Wang

**Affiliations:** Department of Neurology, The First affiliated Hospital of Harbin Medical University, Harbin, China

## Abstract

**Context:**

It is common practice worldwide for health professionals to study abroad. However, the outcome of such experience has not been rigorously evaluated in China. Our current study aimed to quantify the impact on research of studying abroad among Chinese health professionals.

**Methods:**

A self-administered structured questionnaire was developed among health professionals in Harbin Medical University and its affiliated hospitals who had studied abroad (‘returning’ professionals) and health professionals who did not have experience abroad (‘resident’ professionals). 166 ‘returning’ professionals (Group A) and 166 age-, sex- and specialty-matched ‘resident’ professionals (Group B) were included in the study. SPSS software was used for data entry and analysis.

**Results:**

The total IF of papers published by Group A and Group B was, respectively, 1933.52 and 629.23 (*P* < 0.01) and the number of NSFC was 154 and 34 (*P* < 0.01), respectively. The total IF of papers published abroad was associated with the duration abroad (*P <* 0.01) and not with the age of going abroad (*P* > 0.05). The total IF of papers published at home, and the number of NSFC had no relationship with the duration abroad (both *P* > 0.05) nor the age of going abroad (both *P* > 0.05). The total IF of papers published at home and the number of NSFC were positively correlated with the total IF of papers published abroad (both *P <* 0.01).

**Conclusions:**

This study reflects the beneficial experience of working overseas. The opportunity for overseas experience should not be limited by age. Overseas study should be prolonged.

## Introduction

Globalization and internationalization have become key areas of focus for university educators over the past decade, with an increasing emphasis among universities on global health programs and international experience. Health professional colleges are no exception to the rapid expansion of global health initiatives across university campuses. Many medical programs have incorporated studies on cultural differences and awareness into their curricula, and broadened their opportunities for international training through work, education, and research activities [1-6]. Today, health professionals who have overseas training represent more than a quarter of the medical and nursing workforce of Australia, Canada, the UK and the USA [7]. Engagement in global health programs provide significant benefits to health professionals, including an ability to appreciate cultural diversity, the capacity to adapt to societal change, knowledge of alternative approaches to health and disease, and an understanding of public health and its implications for underserved populations [8-10].

According to the National Health Human Resources Survey in China in 2005, only 0.18% of all health professionals had experienced more than 6 months study or training abroad [11]. With the aim of improving healthcare, Harbin Medical University (HMU) and affiliated hospitals in Heilongjiang Province, China have developed an international academic collaborative exchange program in clinical medicine and public health. This study aimed to compare the publishing history of health professionals who had overseas study or training experience (‘returning’ professionals) with that of health professionals who did not have experience abroad (‘resident’ professionals). We hope that the findings of this study will help to evaluate the impact of overseas experience on research capacity of Chinese health professionals and indicate how medical education policies can be improved.

## Methods

There are two medical schools in Harbin: Harbin Medical University (HMU) and Heilongjiang University of Chinese Medicine (HUCM). HMU and HUCM are the statutory regulatory and registration authorities, respectively, for Western medicine and Chinese medicine in Harbin. Most medical professionals of HUCM do not have the opportunity to study abroad, as Chinese medicine is only taught in China, and if they do go overseas, they tend to teach techniques such as acupuncture and massage. Thus, we excluded HUCM from our study.

HMU is a government-owned institution that offers a 5-year medical program leading to a Bachelor degree, a 3-year program leading to a Master degree and a 3-year program leading to a PhD degree, and has strived to restructure its medical education program to offer comprehensive solutions to national health care problems. There has been a trend of studying or training abroad among health professionals from HMU and its four affiliated hospitals, with around 20% of its approximately 4500 professional staffs having overseas experience.

This study was performed from September 2012 to June 2013. Since less than 6-month research training abroad is pretty short and end up insufficient to be a dependent researcher, the 166 ‘returning’ health professionals (Group A) involved in our study had a minimum of 6 months experience abroad including 76 from HMU (Group A1) who are scientific researchers and 90 from the affiliated hospitals (Group A2) who are clinicians. The questionnaire in Table 1 was administered to these 166 professionals manually. Response rates were 100%. Three of the investigators distributed the questionnaires to the professionals, to be collected immediately after completion. The questionnaires were distributed and collected to ensure confidentiality and high response rates. Ethical approval was obtained from the Harbin Medical University Research Centre ethics committee. All participants received written and verbal information about the research project before signing a consent form to participate.

**Table 1:** **Questionnaire which was administered to 166 ‘returning’ professionals**

**The questionnaire for Chinese health professionals in Harbin who have studied or trained abroad**	
Name: ______Department: ________Age: ________	
***questions***	***answers***
Studied or trained abroad from (year/month) to______ (year/month) in (country) ______ University.	
How many scientific papers did you publish when you were abroad?	
What were the IFs of these papers? Which ranked author were you?	
How many scientific papers have you published after you returned from abroad?	
What were the IFs of these papers? Which ranked author were you?	
How many National Natural Science Foundation of China grants have you received?	
When did you receive the foundation?	

The National Natural Science Foundation of China (NSFC) has implemented an advanced science funding system including peer review and performance evaluation. The system is comprehensive and strict, with the number of NSFC accurately reflecting capability in scientific research. The Science Citation Index (SCI) is internationally recognized as the most authoritative scientific literature search tool, and we used the NSFC evaluation and the SCI impact factor (IF) as good indicators of scientific and research capability. Because authors contribute differently to a research study and there is no system internationally which could eliminate this difference, we ranked the authors to reflect this difference according to ‘The Promotion System of HMU (2013)’: first author and corresponding author of a paper (IF × 100%), second author of a paper (IF × 50%), and third author and later authors of a paper (IF × 25%) [12].

The following data were collected among 166 ‘returning’ professionals: (1) age; (2) duration of studying or training abroad (months); (3) the age of going abroad; (4) total IF of papers published abroad; (5) total IF of papers published at home; and (6) number of NSFC. Multiple linear regression was used to determine the factors associated with the IF of papers and number of NSFC using SPSS version 19 (SPSS Inc., Chicago, IL, USA). To evaluate the impact of studying or training abroad, we randomly selected 166 ‘resident’ age-, sex- and specialty-matched professionals (Group B) including 76 scientific researchers from HMU (Group B_1_) and 90 clinicians from the affiliated hospitals (Group B_2_) for comparison of the total IF of papers published and the number of NSFC between the ‘returning’ and ‘resident’ groups. The control group (Group B) was selected based on the exact match of age, sex and specialty as Group A. When there were more than one match, we randomly selected one before developing questionnaire in case of arbitrarily selecting matching controls who are less capable than the treated individuals. When there were no exact match, we replaced the ‘returning’ professional by another one which had at least one match. Also, we developed questionnaires among these 166 ‘resident’ professionals to collect data and response rates were 100%. All data were anonymized to protect the respondents’ privacy.

## Results

Of the 166 ‘returning’ professionals (Group A in Table 2), there were 85 male, 81 female, with an age range of 31–70 years (mean, 44.9 years). They had been abroad for 6 to 196 months (mean, 28.2 months). Among these 166 ‘returning’ professionals, there were 76 scientific researchers ( 30 male, 46 female, Group A_1_) with an age range of 31–70 years (mean, 44.7 years) and 90 clinicians (55 male, 35 female, Group A_2_) from affiliated hospitals with an age range of 31–70 years (mean, 45.1 years). Similarly, there were 166 ‘resident’ age-, sex-and specialty-matched professionals ( 85 male, 81 female, Group B in Table 3) with an age range of 31–70 years (mean, 44.9 years) including 76 scientific researchers (30 male, 46 female, Group B_1_) with an age range of 31–70 years (mean, 44.7 years) and 90 clinicians (55 male, 35 female, Group B_2_) with an age range of 31–70 years (mean, 45.1 years).

**Table 2:** **Data of 166 ‘returning’ professionals (Group A), including 76 professionals at HMU (Group A1) and 90 professionals at affiliated hospitals of HMU (Group A2 )**

		**Maximum**		**Minimum**		**Mean**
Age	Group *A*	70	Group *A*	31	Group *A*	44.9
	Group *A*_*1*_	70	Group *A*_*1*_	31	Group *A*_*1*_	44.7
	Group *A*_*2*_	70	Group *A*_*2*_	31	Group *A*_*2*_	45.1
Duration of studying or training abroad (months)	Group *A*	196	Group *A*	6	Group *A*	28.2
	Group *A*_*1*_	196	Group *A*_*1*_	6	Group *A*_*1*_	29.7
	Group *A*_*2*_	84	Group *A*_*2*_	6	Group *A*_*2*_	27.0
Age of going abroad	Group *A*	55	Group *A*	25	Group *A*	35.3
	Group *A*_*1*_	47	Group *A*_*1*_	25	Group *A*_*1*_	36.0
	Group *A*_*2*_	55	Group *A*_*2*_	25	Group *A*_*2*_	34.7
Total IF of papers published abroad	Group *A*	94.69	Group *A*	0.00	Group *A*	3.83
	Group *A*_*1*_	94.69	Group *A*_*1*_	0.00	Group *A*_*1*_	4.58
	Group *A*_*2*_	60.22	Group *A*_*2*_	0.00	Group *A*_*2*_	7.32
Total IF of papers published at home	Group *A*	113.07	Group *A*	0.00	Group *A*	11.65
	Group *A*_*1*_	113.07	Group *A*_*1*_	0.00	Group *A*_*1*_	17.16
	Group *A*_*2*_	55.00	Group *A*_*2*_	0.00	Group *A*_*2*_	6.99
Number of NSFC	Group *A*	7	Group *A*	0	Group *A*	0.93
	Group *A*_*1*_	7	Group *A*_*1*_	0	Group *A*_*1*_	1.21
	Group *A*_*2*_	6	Group *A*_*2*_	0	Group *A*_*2*_	0.69

**Table 3:** **Data of 166 ‘resident’ professionals (Group B), including 76 professionals at HMU (Group B1) and 90 professionals at affiliated hospitals of HMU (Group B2) at affiliated hospitals of HMU**

		**Maximum**		**Minimum**		**Mean**
Age	Group *B*	70	Group *B*	31	Group *B*	44.9
	Group *B*_*1*_	70	Group *B*_*1*_	31	Group *B*_*1*_	44.7
	Group *B*_*2*_	70	Group *B*_*2*_	31	Group *B*_*2*_	45.1
Total IF of papers	Group *B*	72.03	Group *B*	0.00	Group *B*	3.79
	Group *B*_*1*_	72.03	Group *B*_*1*_	0.00	Group *B*_*1*_	5.75
	Group *B*_*2*_	29.16	Group *B*_*2*_	0.00	Group *B*_*2*_	2.13
Number of NSFC	Group *B*	5	Group *B*	0	Group *B*	0.20
	Group *B*_*1*_	5	Group *B*_*1*_	0	Group *B*_*1*_	0.39
	Group *B*_*2*_	1	Group *B*_*2*_	0	Group *B*_*2*_	0.04

**Figure 1:**
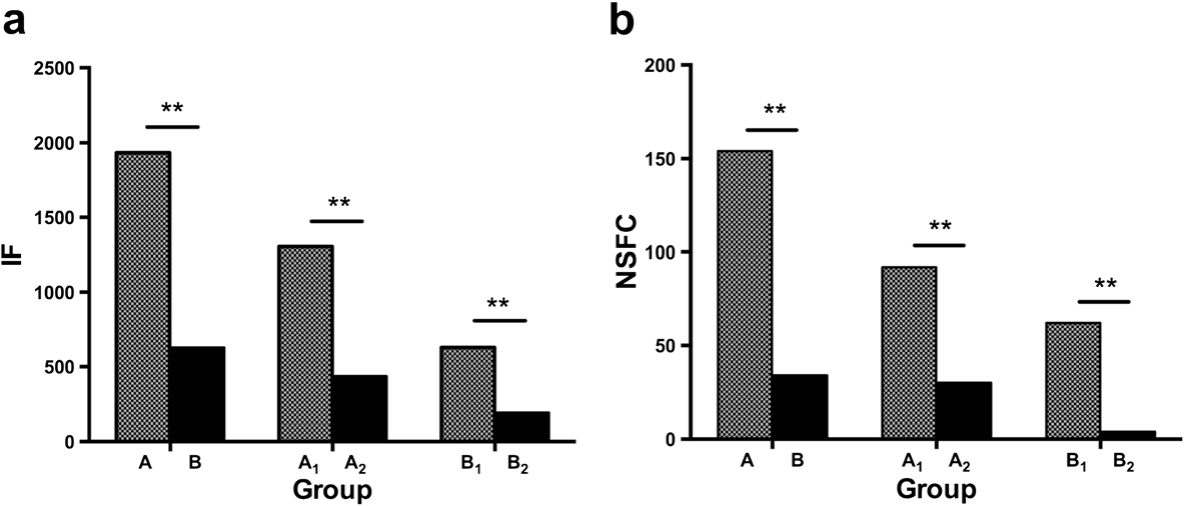
**Total IF and NSFC of ‘resident’ and ‘returning’ professionals.** **(a)** Total IF of ‘resident’ and ‘returning’ professionals. **(b)** NSFC of ‘resident’ and ‘returning’ professionals. Group A/A_1_/A_2_: ‘returning’ professionals; Group B/B_1_/B_2_: ‘resident’ professionals. NSFC: National Natural Science Foundation of China. For 3 paired samples (Group A and Group B, Group A_1_ and Group B_1_ and Group A_2_ and Group B_2_) statistical differences were found for total IF of papers (**P < 0.01) and the number of NSFC (**P < 0.01).

The IF of papers published individually at home of ‘returning’ professionals (Group A in Table 2) ranged from 0.00 to 113.07 (mean, 11.65) and the number of NSFC ranged from 0 to 7 (mean, 0.93). The total IF of papers published at home by Group A was 1933.52 and the number of NSFC was 154. The total IF of papers published at home by Group A_1_ and Group A_2_ was 1304.28 and 629.24, respectively. The number of NSFC of Group A_1_ and Group A_2_ was 92 and 62, respectively. In contrast, the IF of per individual in ‘resident’ professionals (Group B in Table 3) ranged from 0.00 to 72.03 (mean, 3.79) and the number of NSFC ranged from 0 to 5 (mean, 0.20). The total IF of papers published by Group B was 629.23 and the number of NSFC was 34. The total IF of papers published by Group B_1_ and Group B_2_ was 437.13 and 192.10, respectively. The number of NSFC of Group B_1_ and Group B_2_ was 30 and 4, respectively. We compared the mean scores using *t*-test for 3 paired samples (Group A and Group B, Group A_1_ and Group B_1_ and Group A_2_ and Group B_2_ in Figure 1). For Group A and Group B statistical differences were found for total IF of papers (*P* < 0.01) and the number of NSFC (*P* < 0.01). There were significant differences between Group A_1_ and Group B_1_ in mean scores for total IF of papers (*P* < 0.01) and the number of NSFC (*P* < 0.01). The same situation applied to Group A_2_ and Group B_2_.

Analyzing the data in Table 2 of the 166 ‘returning’ professionals using multiple linear regression, we came to a conclusion that the total IF of papers published abroad was related to the duration of studying or training abroad (*P <* 0.01) and not to the age at which the subjects went abroad (*P >* 0.05). The total IF of papers published at home and number of NSFC had no association with the duration of studying or training abroad (both *P >* 0.05) nor with the age of going abroad (both *P >* 0.05). However, the total IF of papers published at home and the number of NSFC were positively correlated with the total IF of papers published abroad (both *P <* 0.01).

## Discussion

It is often supposed that studying or training abroad has a positive influence on health professionals’ capacity for scientific research. However, the impact has not been evaluated in Chinese health professionals. This study revealed that there is a wide gap in publishing history between ‘returning’ and ‘resident’ health professionals including both scientific researchers and clinicians. These data strongly suggest that not only scientific researchers but also clinicians could gain much benefit from a period of study or training abroad by broadening their horizons and boosting their capability in scientific research.

So what can be done to promote this positive influence? Traditionally, neither legislation nor other measures have been taken to optimize the ‘overseas experience’ of health care [13]. It is important for clinical faculties and scientists, and administrators and government officials engaged in health care to examine and integrate resources to increase overseas training and help improve home research capacity. Our findings indicate that the total IF of papers published at home and the number of NSFC was associated with the total IF of papers published abroad, which was related to the duration of studying or training abroad, and not the age of going abroad. These results suggest that the opportunity for overseas experience should not be limited by age. Based on this incentive result, the principles that older professors should not be considered to be appropriate persons for going abroad could be abolished. The ‘returning’ professionals should focus on making an effort to improve the total IF of papers published abroad by prolonging the duration of study abroad, perhaps by studying for a PhD or other higher degree.

Another area that requires special attention is the quality of research facilities in universities which professionals attend abroad. It is widely recognized that health professionals cannot publish a paper with a high IF unless the laboratory equipment and facilities are of a high standard. We suggest that a quality control system be developed to ensure that health professionals going abroad have access to institutions offering fully equipped up-to-date laboratories. This would provide ‘returning’ professionals with excellent research facilities and knowledge about the newest procedures.

During conversations with ‘returning’ professionals, we realized that there were major differences in the research conditions between China and abroad, and this appeared to be the main factor that motivated health professionals to study abroad. Not only do poor research conditions at home hamper innovative thinking, but they also reduce the capacity for study implementation. This situation can only be improved by very strong political will and greater budget allocations to the health care sector. According to the Ministry of Finance, the total expenditure on health care in 2012 was just 6% of the gross domestic product in China. It is necessary that the political hierarchy take some bold steps in this regard. However, this is not only a governmental responsibility, and the medical workforce working across China should also address all the factors that lead to the poor research capability of health professionals. Legislation should be put in place to ensure that the working environment is made more conducive to medical research. Only then will we be able to improve the national capability in research.

### Study limitations

Our work represents an initial effort to try to better understand the feasibility and outcomes of studying or training abroad among health professionals in Harbin. This study involved only one medical school and its four affiliated hospitals and thus had a small sample size; therefore it does not represent the entire medical workforce in China. Since authors contribute differently to a research study and there is no author ranking system internationally which could eliminate the difference among authors, we ranked the authors according to ‘The Promotion System of HMU (2013)’. This system could not be applied to all SCI papers worldwide. Moreover, there are some major confounders, for example, language proficiency, funds, facility, equipment, job titles and specialties. Those who get 6 months or more of international training are likely to be selected out of motivated candidates, so they may have higher capability and motivation at baseline (before getting exposed to international training). So there may be deviation to compare those with and without international experience unless the authors’ capability to conduct research and write papers at baseline is matched by quantified standards.

## Conclusions

This study shows the major positive impact on medical research in China of a period of studying or training abroad, and indicates that the opportunity for overseas experience should not be limited by age. The ‘returning’ professionals should focus on making an effort to improve the total IF of papers published abroad by prolonging the duration of study abroad. The study also indicates that there is a strong need for upgrading research facilities in China.

## Abbreviations

HMU: Harbin Medical University
HUCM: Heilongjiang University of Chinese Medicine
NSFC: National Natural Science Foundation of China
SCI: Science citation index
IF: Impact factor
